# Acute L-arginine alpha ketoglutarate supplementation fails to improve muscular performance in resistance trained and untrained men

**DOI:** 10.1186/1550-2783-9-17

**Published:** 2012-04-17

**Authors:** Benjamin Wax, Andreas N Kavazis, Heather E Webb, Stanley P Brown

**Affiliations:** 1 Kinesiology Department, Mississippi State University, P.O. Box 6186, Mississippi State, 39762, USA

**Keywords:** Resistance exercise, Ergogenic aids, Nitric oxide

## Abstract

****Background**:**

Dietary supplements containing L-arginine are marketed to improve exercise performance, but the efficacy of such supplements is not clear. Therefore, this study examined the efficacy of acute ingestion of L-arginine alpha-ketoglutarate (AAKG) muscular strength and endurance in resistance trained and untrained men.

****Methods**:**

Eight resistance trained and eight untrained healthy males ingested either 3000mg of AAKG or a placebo 45 minutes prior to a resistance exercise protocol in a randomized, double-blind crossover design. One-repetition maximum (1RM) on the standard barbell bench press and leg press were obtained. Upon determination of 1RM, subjects completed repetitions to failure at 60% 1RM on both the standard barbell bench press and leg press. Heart rate was measured pre and post exercise. One week later, subjects ingested the other supplement and performed the identical resistance exercise protocol.

****Results**:**

Our data showed statistical significant differences (p<0.05) between resistance trained and untrained males for both 1RM and total load volume (TLV; multiply 60% of 1RM times the number of repetitions to failure) for the upper body. However, 1RM and TLV were not statistically different (p>0.05) between supplementation conditions for either resistance trained or untrained men in the bench press or leg press exercises. Heart rate was similar at the end of the upper and lower body bouts of resistance exercise with AAKG vs. placebo.

****Conclusion**:**

The results from our study indicate that acute AAKG supplementation provides no ergogenic benefit on 1RM or TLV as measured by the standard barbell bench press and leg press, regardless of the subjects training status.

## **Background**

The use of nutritional supplements has exponentially increased in the past decade [1-3]. In particular, supplements containing L-arginine are extremely popular among healthy people engaging in resistance training exercises [4,5]. Generally, these supplements are marketed as nitric oxide stimulators, which purpose to increase muscular strength and endurance as potential benefits to the user. The premise of these claims are that they increase the availability of arginine in the system, thus augmenting synthesis of nitric oxide release by way of the enzyme nitric oxide synthase [4,6,7].

It is believed that this increase in nitric oxide will allow for improved blood flow [8,9] and this could potentially be beneficial for individuals performing resistance exercises. Further, an elevation in blood flow could theoretically improve exercise performance by increasing nutrient delivery and/or waste-product removal from exercising skeletal muscles [10-12]. It should be noted that concentrations of L-arginine in the body can be the rate limiting step for nitric oxide production [7,13,14]. However, there is still no clear evidence to conclude L-arginines role as a nitric oxide stimulator that improves resistance exercise performance in healthy adults [4].

Recently, commercially available L-arginine supplements have been combined with alpha ketoglutarate, in an effort to further improve exercise performance by increasing adenosine triphosphate production through the electron transport chain [15]. Specifically, alpha ketoglutarate is a metabolite produced by the oxidative decarboxylation of isocitrate; a process that occurs in the Krebs cycle [13,16]. An exogenous supply of alpha ketoglutarate through a supplement such as L-arginine alpha-ketoglutarate (AAKG) could increase Krebs cycle flux thus increasing the rate acetyl-CoA oxidation [15]. Furthermore, supplementation with alpha ketoglutarate may have a glutamate sparing effect in the body. This is important as alpha ketoglutarate can be replenished through the transamination of glutamate [17], which is an amino acid necessary for protein anabolism and it is also known to be a very important excitatory nervous system neurotransmitter [18,19]. Thus, supplementation with alpha ketoglutarate may have both neurological and metabolic ergogenic properties.

Arginine-based supplementation has produced mixed results with some studies reporting ergogenic benefits in anaerobic power [13], muscular strength [13,20], and muscular endurance [21], while others have found no effect on these same performance variables [22,23]. Specifically, Santos et al. reported decreased muscular fatigue following L-arginine ingestion [24], while Greer and Jones reported no ergogenic benefits during muscular endurance exercises [22]. To our knowledge, only two studies have investigated the independent effects of AAKG on resistance exercise performance [13,22]. Therefore, the aim of this study was to investigate the ergogenic properties of acute AAKG ingestion in untrained and resistance trained men on measures of upper and lower body one-repetition maximum (1RM) strength and total load volume (TLV).

## **Methods**

### **Subjects**

Sixteen apparently healthy males participated in the study. Eight participants (19.81.9years, 1.760.09m, 78.17.5kg) had been engaged in resistance exercise training (at least two times per week for the past six months) and these men were classified as the resistance trained subjects of the study. The eight remaining participants had not engaged in resistance training for the prior three years (21.82.4years, 1.790.04m, 88.622.4kg) and these men were classified as the untrained subjects in the study. Prior to the study, subjects completed a health history questionnaire and signed a statement of informed consent. All experimental procedures were reviewed and approved by the Institutional Review Board at Mississippi State University prior to the initiation of the study.

### **Experimental approach to the problem**

Each subject reported to the laboratory three times at the same time of the day. The first session was used to determine subjects anthropometric data and served as a familiarization session for the exercise protocol. Subjects were instructed to refrain from strenuous resistance exercise activities for 48 hours before sessions 2 and 3. Also, subjects were instructed to avoid caffeine and alcohol consumption during the 24 hour period preceding sessions 2 and 3. All subjects reported complying with these guidelines.

A randomized, counterbalanced, double blind design was used for this study. The protocol employed in this study was based on a prior study [25], which tested the efficacy of a proposed ergogenic supplement on muscular force and endurance. In the current study, subjects ingested either 3000mg of AAKG or placebo prior to measures of upper and lower body 1RM strength and TLV. One week later, subjects ingested the other supplement and performed the same exercise protocol. A one-week interval was utilized to ensure muscle recovery and allow clearance of the supplement from the body. In order to investigate the ergogenic benefits of acute AAKG on exercise performance the following dependent measures were obtained: HR, 1RM strength and TLV for upper and lower body.

### **Supplementation**

Participants arrived at the lab and were asked about their activity level for the preceding 48 hours. Upon clearance and following a 5 minute rest, the participant ingested either a serving of commercially available AAKG (Healthwatchers DE Inc., Bohemia, NY) or a placebo composed of microcrystalline cellulose (Apotheca Inc., Woodbine, IA) with 300ml of water. The placebo was similar in color, size, and texture to the supplement. The selected dose [26] and the timing of supplementation was based on prior research which reported plasma arginine concentrations peaked approximately 60 minutes following oral ingestion of AAKG [27]. Because of budgetary constraints, the ingredients of the supplement were not confirmed via an independent laboratory analysis, and consequently, quality control could be a confounding factor.

### **Exercise protocol**

The subjects then rested quietly for 45 minutes following the ingestion of the supplement. Next, subjects warmed up on an upright stationary bike (Life Fitness, Brunswick Corporation, Lake Fores, IL) for 5 minutes. Then, subjects completed two warm-up sets of 1012 repetitions on the standard barbell bench press (Magnum D78, Magnum Fitness Systems, South Milwaukee, WI) with a 61.2kg mass. In order to determine each subjects 1RM on the bench press, a trained technician determined a beginning resistance for the subject to perform their first 1RM trial. One-repetition maximum was then determined by increasing mass in 4.5 to 9.1kg increments relative to the subjects ability to lift the first weight. The 1RM was obtained in three to six sets for all subjects. The accepted 1RM was defined as the ability of the subject to complete a full repetition without assistance. Following a three minute rest period, 60% of 1RM was placed on the standard barbell bench press and each subject completed as many repetitions as possible until failure occurred. Failure was defined as the inability to complete a full repetition without assistance. Total load volume for the upper body was calculated by multiplying the 60% of the 1RM by the number of repetitions to failure.

Following a five minute rest period, subjects performed two warm-up sets (1215 repetitions) of leg press on a Cybex 45 plate loaded leg press (Cybex Inc., Medway, MA) at a load of 82kg. A trained technician then determined a beginning resistance for the participant to perform their first 1RM trial. One-repetition maximum was then determined by increasing mass in 9.1 to 18.1kg increments relative to the participants ability to lift the first weight. The 1RM was obtained in three to six sets with the same criteria described earlier. Following a three minute rest period, 60% of 1RM was placed on the leg press and each participant completed as many repetitions as possible until failure occurred and TLV for lower body was calculated according to the previously described method.

Heart rate was measured at rest (pre) and within 5 seconds of the final repetition following upper body (post upper) and lower body (post lower) failure by using an automated instrument (SunTech Medical, Morrisville, NC).

Seven days after the completion of session 2, subjects ingested the other supplement and repeated the identical protocol. Importantly, based on information reported by subjects, pre-testing (no strenuous resistance exercise 48 hours before testing, well hydrated, sufficient sleep, etc) and testing conditions (e.g. time of day, arousal, etc) were similar between session 2 and session 3.

### **Statistical analyses**

All statistical analyses were performed by using the GraphPad Prism (GraphPad Software, Inc., La Jolla, CA). A sample size analysis was performed and showed that at least eight subjects were required in each group to achieve a power of 0.80. Data for 1RM and TLV between AAKG and placebo were analyzed using a 2 (condition; AAKG or placebo) x 2 (status; untrained or trained) repeated measures analysis of variance (ANOVA) followed by an independent t-test when the 2 x 2 ANOVA resulted in significant difference. Data for HR were analyzed by using a 2 (condition; AAKG or placebo) x 3 (time; pre, post upper, post lower) repeated measures ANOVA, followed by paired t-test when the 2 x 3 ANOVA resulted in significant difference. Statistical significance was established at p<0.05. Data are reported as meanstandard deviation.

## **Results**

All 16 subjects who initially volunteered completed the testing procedures. There was no order effects observed between the 2 trials (p>0.05).

**Figure 1:**
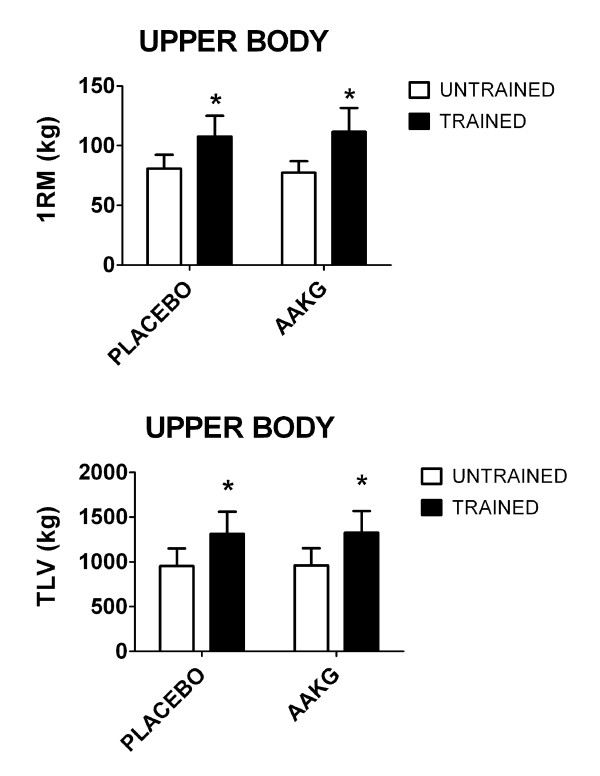
**One-repetition maximum (1RM) and total load volume (TLV=60% of one-repetition maximum X repetitions to failure) on the bench press.** Data are presented as meanstandard deviation. * indicates p<0.05 between untrained and trained subjects during same condition (placebo or L-arginine Alpha-Ketoglutarate (AAKG)).

Comparison of resistance trained and untrained subjects demonstrated trained subjects had statistically significantly higher (p<0.05) 1RM and TLV (Figure 1) than untrained subjects for upper body under both supplementation conditions (i.e. AAKG and placebo). We did not observe a significant difference (p>0.05) in 1RM or TLV when comparing AAKG and placebo supplementation in either resistance trained or untrained subjects.

**Figure 2:**
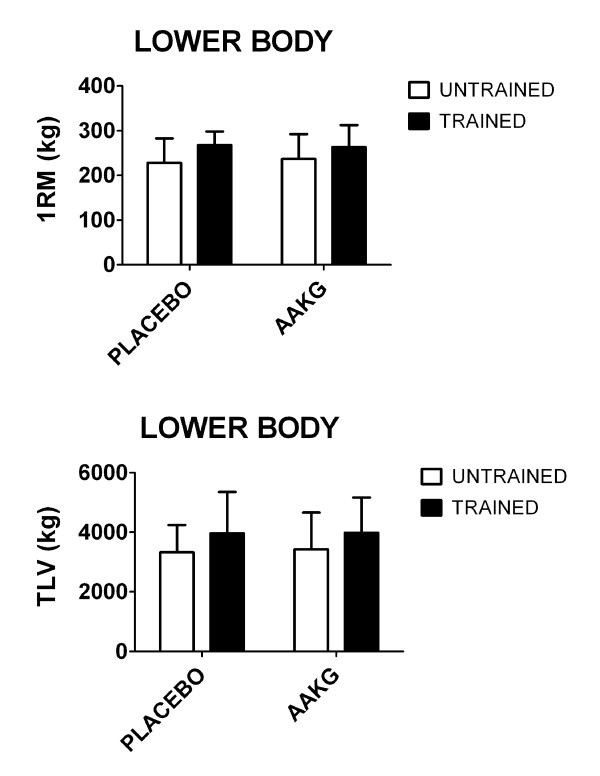
**One-repetition maximum (1RM) and total load volume (TLV=60% of one-repetition maximum X repetitions to failure) on the leg press.** Data are presented as meanstandard deviation. AAKG=L-arginine Alpha-Ketoglutarate.

In regards to 1RM and total load volume of the lower body we do not observe any significant differences (p>0.05), regardless of the supplementation conditions (i.e. AAKG and placebo) or training status (trained and untrained) (Figure 2).

**Figure 3:**
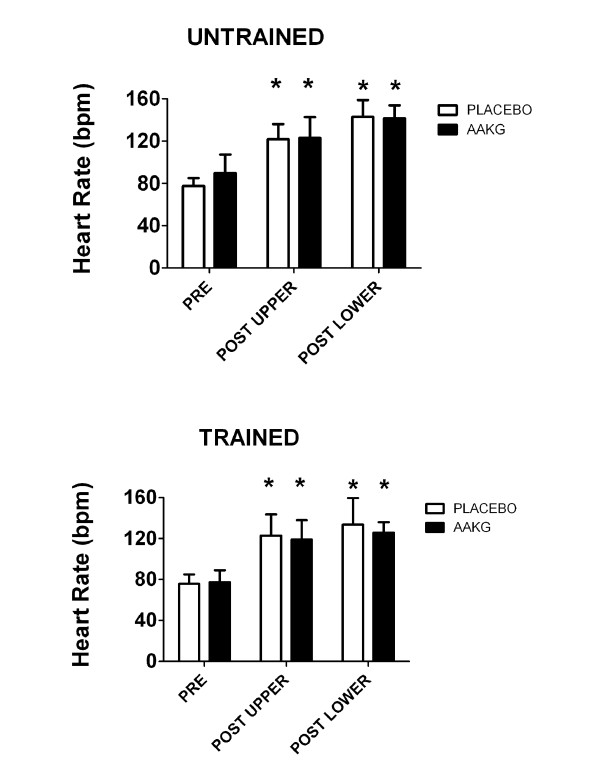
**Heart rate (beats per minute; bpm) in untrained and trained subjects at PRE (i.e. rest), POST UPPER (i.e., following bench press protocol), and POST LOWER (i.e., following leg press protocol).** * indicates p<0.05 compared to PRE. AAKG=L-arginine Alpha-Ketoglutarate.

Heart rate was measured as an indicator of exercise intensity and to document that subjects exerted similar effort following placebo and AAKG supplementation. The 2 x 3 ANOVA for HR responses demonstrated no interaction effects, but a main effect for time was revealed (p<0.05). Post-hocs demonstrated increases in HR after the upper body and lower body compared to rest, although there was were differences between conditions (AAKG and placebo) (Figure 3).

## **Discussion**

The major finding of this study was that an acute ingestion of 3000mg of AAKG had no effect on upper or lower body 1RM or TLV in either resistance trained or untrained men. The ergogenic benefits of arginine-based supplementation remain equivocal in the literature.

Some authors have reported increases in anaerobic performance [13,20] and muscular endurance [21] after ingesting arginine-based supplements. However, like our current study, Greer and Jones [22] did not find an ergogenic effect on exercise performance variables following acute ingestion of AAKG. This may suggest that a specific loading period may be necessary for the prospective ergogenic effects of arginine-based supplements to be realized. Specifically, Santos et al. [21] observed a significant increase in muscular endurance after 15days of oral supplementation with L-arginine aspartate (3g/day), while Campbell et al [13] reported significant increases in maximal strength and anaerobic power following 8weeks of oral supplementation with AAKG (6g of L-arginine and 6g of alpha-ketoglutarate). These authors did not investigate the underlying mechanism that contributed to the positive effects following chronic L-arginine supplementation; however, speculation regarding increased coronary and peripheral blood flow because of inhibition of endothelin has been proposed [28].

Heart rate increases linearly as exercise intensity increases [29-32] and well documented response of HR can be used as an indicator of exercise intensity [33,34]. While the present findings reflect this relationship, HR values were not significantly different between subjects that ingested AAKG or placebo. This observation was the same regardless of the training status of the subjects. Furthermore, data obtain from this study are in agreement with previous investigations, which reported that supplementation with nitric-oxide-inducing supplements had no effect on the resting levels of HR [5,35]. However, these findings have not been consistent. Specifically, it has been reported that arginine-based supplementation did not have a large influence on hemodynamics in healthy humans following an exercise protocol that lasted twelve minutes [36]. In agreement with these data, Bloomer and collaborators have also reported that HR was not altered after single bouts of anaerobic or resistance exercise following ingestion of nitric-oxide inducing supplements [35]. Conversely, a different study reported increases in HR following the ingestion of an arginine-based supplement on single bouts of resistance exercise [5]. These variable responses of HR following exercise may be due to exercise protocol selection and/or amount of muscle mass recruited during exercise.

There are also general limitations with this study. Firstly, an acute exogenous dose of AAKG may not be sufficient to facilitate the increased levels of arginine necessary to confer an ergogenic effect in normal healthy individuals [37]. Previous research has demonstrated that following ingestion, nearly 50% of oral arginine-based supplements are metabolized by the enterocytes and the liver [38], thus, a longer loading phase may be required. Secondly, in contrast to previous studies utilizing repeated bouts of exercise, we examined the efficacy of administering one lone AAKG dose prior to a 1RM test and a single bout of exercise (60% of 1RM) to failure and observed no difference in resistance exercise performance attributable to AAKG. The use of a single-bout condition was selected in response to a prior study which reported significant differences in subjects 1RM following AAKG supplementation [13]. Finally, while there was a significant difference between the two groups (resistance trained and untrained) in upper body strength, lower body strength differences among trained and untrained men did not reach significance. Therefore, it would have been more prudent to classify groups based on strength differences, not self reported training status.

Finally, a very important issue to consider when people orally ingest prolonged types of L-arginine supplementation (> 7days) is the potential for adverse events to occur. In this regard, a recent paper reported that individuals had experienced adverse side effects following ingestion of nitric oxide stimulator supplements [39]. However, other investigators (as in the current study) have reported that acute ingestion of AAKG ingestion appears to be safe and well tolerated in healthy subjects [13].

## **Conclusion**

Arginine-based supplements, such as AAKG, are marketed as nitric oxide stimulators since nitric oxide can be endogenously synthesized from L-arginine. An increase in nitric oxide could theoretically improve exercise performance by increasing nutrient delivery and/or waste-product removal from exercising skeletal muscles. However, in the current study, acute AAKG supplementation provided no ergogenic benefit, regardless of the subjects training status. Based on the current study an acute ingestion of AAKG is not recommended for healthy individuals to increase maximal strength and muscular endurance for resistance training exercises.

## **Competing interests**

The authors (BW, ANK, HEW, and SPB) declare that they have no competing interests.

## **Authors contributions**

BW, ANK and HEW were responsible the study design, coordination of the study, oversight of data collection and analysis. SPB assisted in manuscript preparation. All authors read and approved the final manuscript.
